# Maxillofacial Squamous Cell Carcinoma With Cervical Metastasis and Tuberculous Lymphadenitis: Diagnostic Dilemmas and Clinical Insights

**DOI:** 10.1002/cnr2.70454

**Published:** 2026-01-04

**Authors:** Xierzhati Tuerxun, Meiheriban Tuerhong, Zainure Wubulihasimu, Baihetiyaer Yimin, Maimaitituxun Tuerdi, Kai Liu

**Affiliations:** ^1^ Department of Oral and Maxillofacial Surgery, Kashi Prefecture Second People's Hospital Kashi Xinjiang China; ^2^ Department of Oral and Maxillofacial Trauma and Orthognathic Surgery The First Affiliated Hospital of Xinjiang Medical University Urumqi Xinjiang China; ^3^ Department of Oral and Craniomaxillofacial Surgery, Shanghai Ninth People's Hospital Shanghai Jiao Tong University School of Medicine Shanghai China; ^4^ College of Stomatology Shanghai Jiao Tong University Shanghai China; ^5^ National Center for Stomatology Shanghai China; ^6^ National Clinical Research Center for Oral Diseases; Shanghai Key Laboratory of Stomatology Shanghai China

**Keywords:** coexisting pathology, diagnostic dilemma, neck dissection, squamous cell carcinoma, tuberculous lymphadenitis

## Abstract

**Background:**

Maxillofacial squamous cell carcinoma (SCC) typically metastasizes to lymph nodes, yet coexisting tuberculous lymphadenitis is extraordinarily rare, posing diagnostic challenges.

**Case:**

A 68‐year‐old female with right maxillofacial SCC and ipsilateral lymphadenopathy underwent radical resection and selective neck dissection after computed tomography had shown nodes with central necrosis and rim enhancement—features indeterminate for metastasis versus infection. Histopathological examination of the dissected lymph nodes revealed concurrent metastatic SCC foci and tuberculous granulomas. Further tuberculosis‐specific tests returned positive results, confirming the final diagnosis of metastatic maxillofacial SCC with coexisting tuberculous lymphadenitis. Notably, the patient had no tuberculosis‐related symptoms, with tuberculous lymphadenitis unsuspected preoperatively, underscoring the diagnostic challenge of such coexisting conditions.

**Conclusions:**

This case highlights the importance of considering infectious comorbidities, particularly in cases with atypical imaging or clinical manifestations, when evaluating lymph node lesions in cancer patients to avoid misdiagnosis and optimize therapeutic strategies.

## 1 Introduction

Cutaneous squamous cell carcinoma (SCC) of the maxillofacial region is one of the most common malignant tumors with a high propensity for regional lymph node metastasis [1]. In this case, the patient presented with right facial SCC and ipsilateral cervical lymphadenopathy. Preoperative computed tomography (CT) imaging revealed lymph nodes with rim enhancement and central necrosis, initially suggestive of metastatic involvement. However, histopathological examination unexpectedly identified concurrent metastatic SCC foci and classic tuberculous lesions within the same regional lymph nodes.

In clinical practice, the coexistence of regional lymph node metastasis and tuberculous lymphadenitis is exceedingly rare. Tuberculosis, a chronic granulomatous disease caused by *Mycobacterium tuberculosis* infection, typically results from the dissemination of primary infection or reactivation of latent infection [2, 3]. Notably, immunocompromised states in cancer patients may increase the risk of tuberculosis infection or reactivation of latent infection [4, 5].

A critical diagnostic challenge lies in distinguishing metastatic SCC from tuberculous lymphadenitis: both may present as painless lymphadenopathy with overlapping imaging features (e.g., rim enhancement, central necrosis) [6]. Furthermore, the patient exhibited no tuberculosis‐related symptoms (e.g., low‐grade fever, night sweats), making initial misdiagnosis as isolated metastatic disease highly likely.

This study underscores the necessity of including infectious etiologies—particularly tuberculous lymphadenitis in endemic regions—in the differential diagnosis of lymphadenopathy in cancer patients. Meticulous histopathological evaluation is paramount for diagnosing such complex cases. Misdiagnosis may lead to tuberculosis dissemination during antitumor therapy or inappropriate use of immunomodulatory agents. Through systematic analysis of this case's clinical features, imaging findings, and pathological results, we aim to enhance clinicians' awareness of such complexities and provide insights for optimizing diagnostic strategies.

## 2 Case Presentation

**FIGURE 1:**
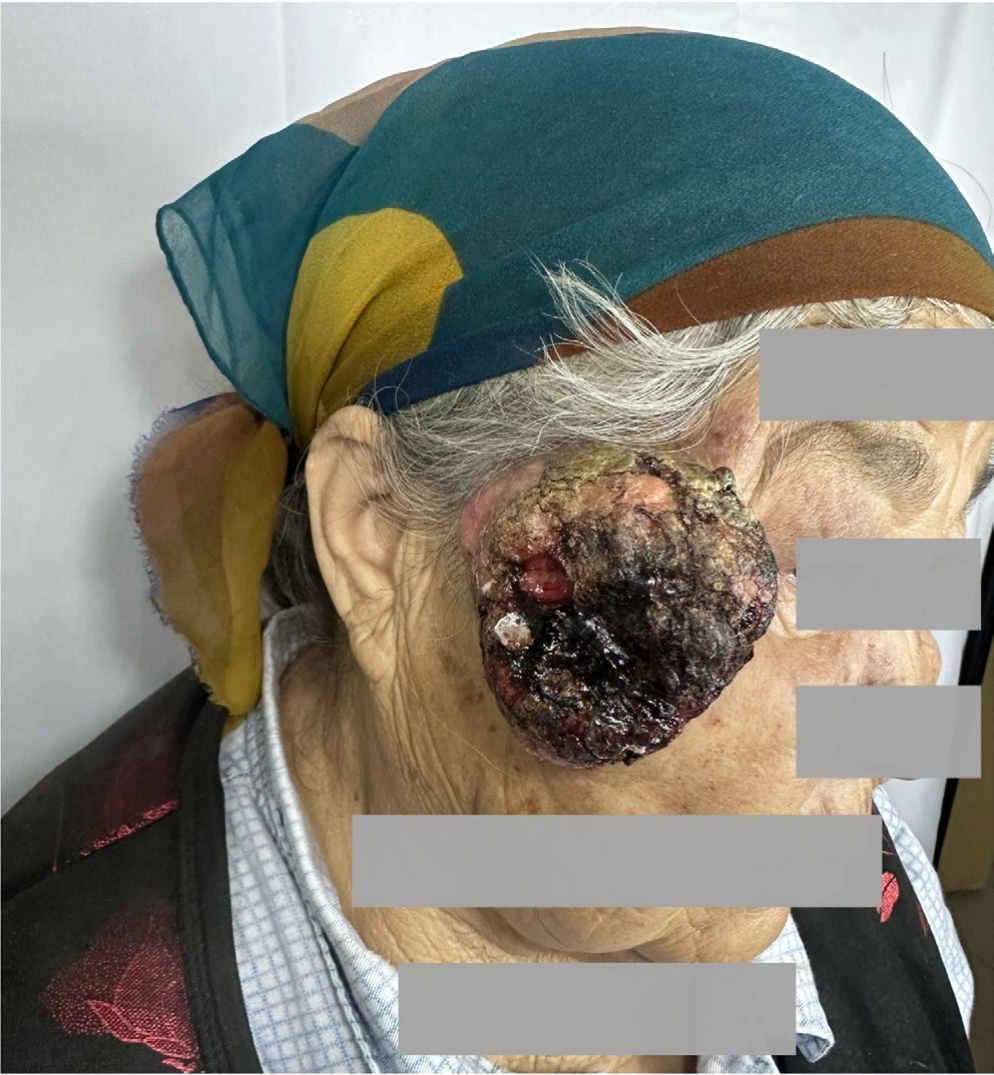
Clinical presentation of the right maxillofacial mass.

A 68‐year‐old female presented to the Department of Oral and Maxillofacial Surgery at the Kashi Prefecture Second People's Hospital, Xinjiang, in late February 2025, with a three‐month history of a progressively enlarging right maxillofacial mass. Physical examination revealed a firm, irregularly shaped exophytic mass (~6 × 5 × 4 cm) with a verrucous surface and central ulceration in the right maxillofacial region (Figure 1). The lesion demonstrated poor mobility and was fixed to adjacent tissues. Multiple enlarged lymph nodes were palpated in the right cervical region, with the largest (measuring 2.5 cm in diameter) located in level III, exhibiting limited mobility, and mild tenderness.

**FIGURE 2:**
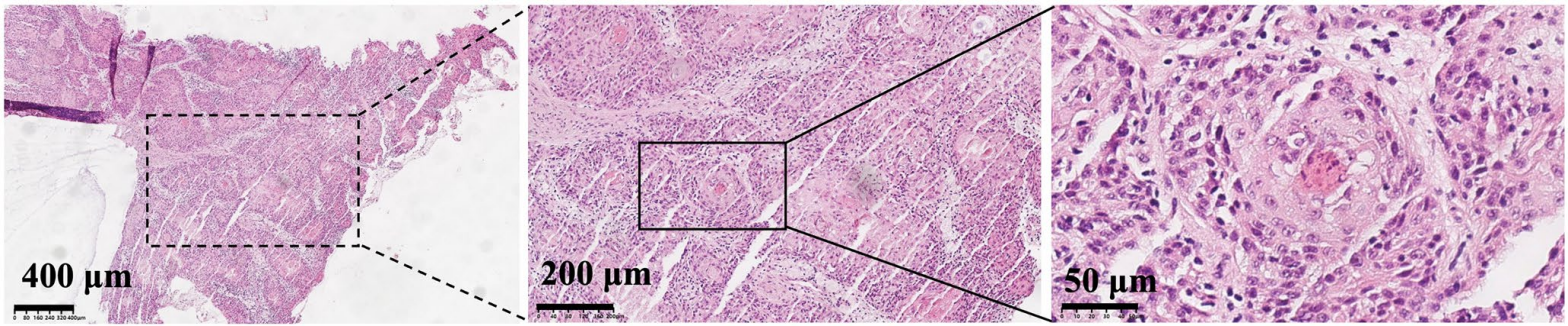
Histopathological features of the preoperative biopsy from the right maxillofacial mass are consistent with SCC (H&E). Images are shown at original magnifications of 4× (scale bar: 400 μm), 10× (scale bar: 200 μm), and 40× (scale bar: 50 μm).

Preoperative biopsy of the right maxillofacial mass confirmed the diagnosis of SCC, demonstrating irregularly arranged nests of atypical squamous cells with keratin pearl formation, as shown in Figure 2.

**FIGURE 3:**
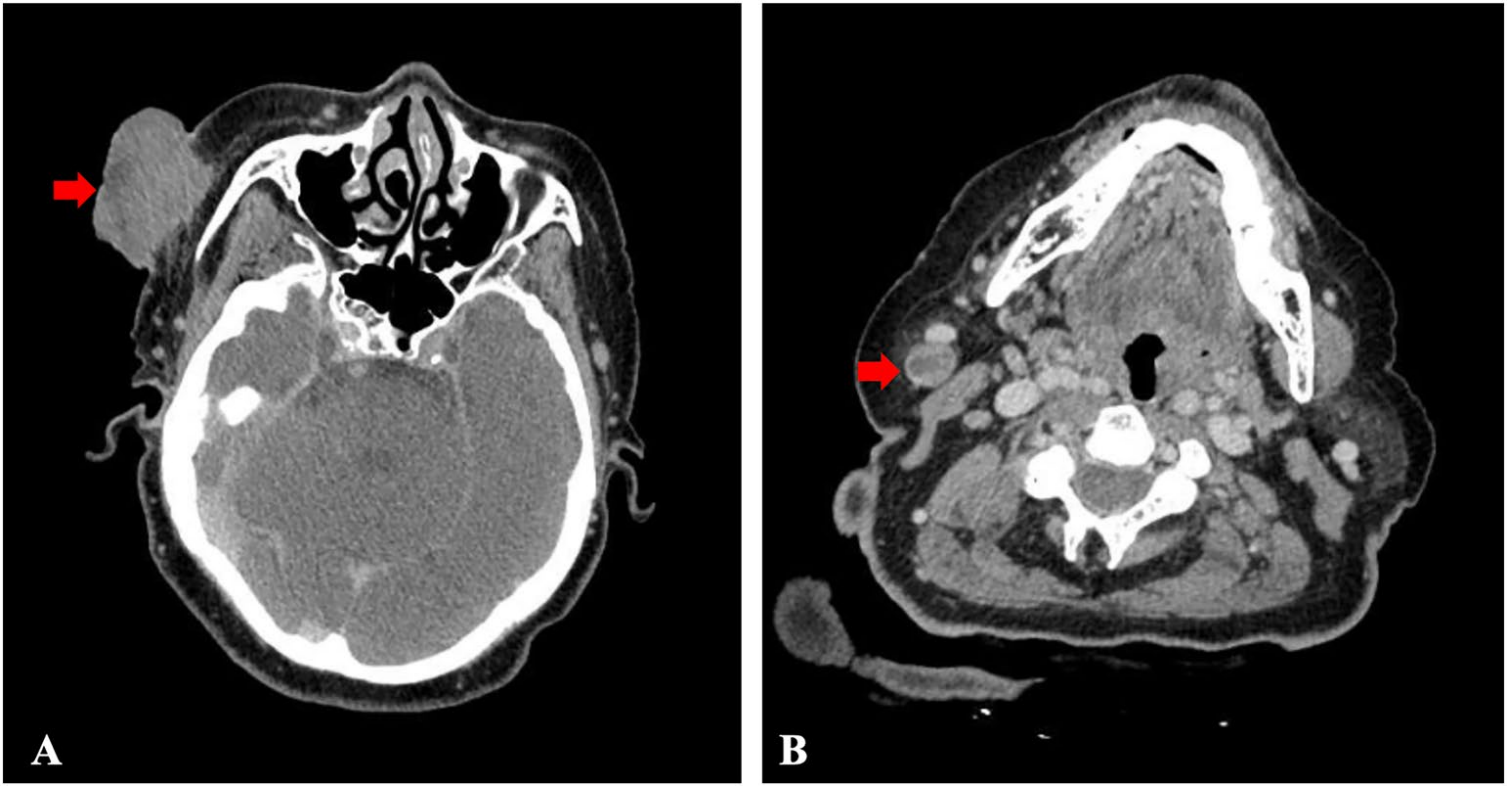
CT manifestations of right maxillofacial lesion and cervical lymphadenopathy: (A) An infiltrative soft tissue mass (arrow) in the right maxillofacial region demonstrating irregular margins and heterogeneous enhancement; (B) Right cervical lymph nodes exhibiting characteristic peripheral rim enhancement (arrowhead) with central hypodensity.

CT imaging revealed an infiltrative mass in the right maxillofacial region (Figure 3A) and multiple pathologically enlarged lymph nodes in the right cervical chain showing characteristic rim enhancement with central necrosis (Figure 3B). Chest CT revealed no evidence of pulmonary metastases or radiographic signs suggestive of active pulmonary tuberculosis.

The patient has no family history of malignant tumors and denies any history of tuberculosis exposure. During the course of illness, no significant systemic symptoms were observed, including persistent fatigue, intermittent low‐grade fever, or night sweats.

Accordingly, based on the Union for International Cancer Control (UICC) TNM classification, the patient was diagnosed with right maxillofacial SCC and was assigned a clinical stage of T3N2bM0 (Stage IVA).

## 3 Laboratory Investigations

Upon admission in early March 2025, laboratory investigations revealed leukopenia (white blood cell count [WBC] 3.4 × 10^9^/L; reference range 4.0–10.0 × 10^9^/L) with a normal neutrophil percentage (65.2%; reference range 40%–75%). Serological testing was negative for human immunodeficiency virus (HIV), hepatitis B virus (HBV), and hepatitis C virus (HCV) infections.

## 4 Surgical Management

On the third day after admission, the patient underwent resection of the right maxillofacial lesion with 1‐cm margins under general anesthesia, combined with right cervical lymph node dissection. The resultant defect was reconstructed utilizing a submental island flap. Perioperative antibiotic prophylaxis with intravenous cefuroxime (1.0 g every 8 h) was administered, beginning 30 min before surgical incision and continuing for 48 h postoperatively. Dexamethasone 5 mg was administered intravenously once daily for 3 days to mitigate postoperative edema.

## 5 Pathological Examination

### 5.1 Primary Maxillofacial Lesion

**FIGURE 4:**
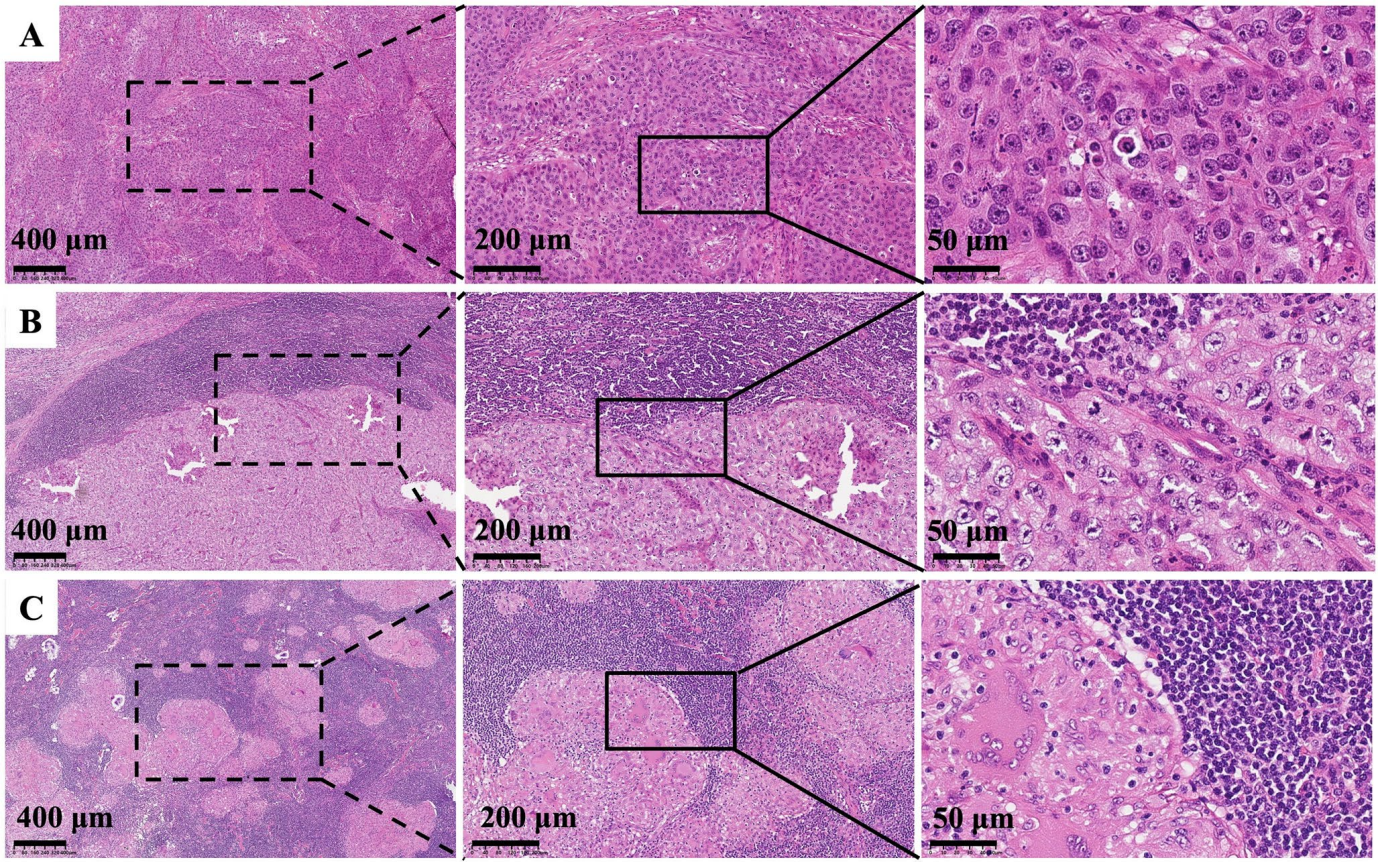
Diagnostic histopathology (H&E): (A) primary SCC; (B) Nodal metastasis showing malignant squamous epithelium; (C) tuberculous lymphadenitis with necrotizing granulomatous inflammation. Images are shown at original magnifications of 4× (scale bar: 400 μm), 10× (scale bar: 200 μm), and 40× (scale bar: 50 μm).

Histopathological features revealed irregularly arranged nests of atypical squamous cells with keratin pearl formation (Figure 4A), corresponding to a pathologic stage of pT3, which was consistent with the clinical T classification. Immunohistochemical analysis demonstrated positive staining for CK5/6, p63, and p53, while p16 was negative. The proliferation index (Ki67) was 10%. Additional markers showed positivity for cytokeratin (CK) and negativity for vimentin.

### 5.2 Lymph Node

#### 5.2.1 Metastatic SCC

The microscopic findings revealed keratin pearl formation, intercellular bridges, and marked atypia (enlarged hyperchromatic nuclei, prominent nucleoli), along with the presence of pathological mitotic figures, confirming metastatic SCC (Figure 4B). Metastatic involvement was present in 2 of 10 resected right cervical lymph nodes, with the largest metastasis measuring 2.5 cm, leading to a pathologic nodal stage of pN2b.

#### 5.2.2 Concurrent Tuberculous Infection

Epithelioid cell granulomas with Langhans giant cells and caseous necrosis, consistent with tuberculous changes, were identified in 5 of the 10 resected right cervical lymph nodes (Figure 4C).

## 6 Supplementary Investigations

As tuberculosis had not been considered in the preoperative differential diagnosis, necrotizing granulomas were identified on postoperative histopathological examination 1 week postoperatively. Consequently, both tuberculin skin testing (TST) and interferon‐gamma release assay (IGRA) were immediately performed, with subsequent results returning positive for both assays.

The patient was ultimately diagnosed with right maxillofacial SCC exhibiting cervical lymph node metastasis, concurrent with tuberculous lymphadenitis. Following negative preoperative screening for HBV/HCV infection (thereby reducing the risk of anti‐tuberculosis drug‐induced hepatotoxicity), standard HRZE regimen was initiated 2 week postoperatively [2]: an intensive phase (2 months) of daily oral isoniazid (5 mg/kg), rifampicin (10 mg/kg), pyrazinamide (25 mg/kg), and ethambutol (15 mg/kg), followed by a continuation phase with isoniazid and rifampicin at the aforementioned dosages.

**FIGURE 5:**
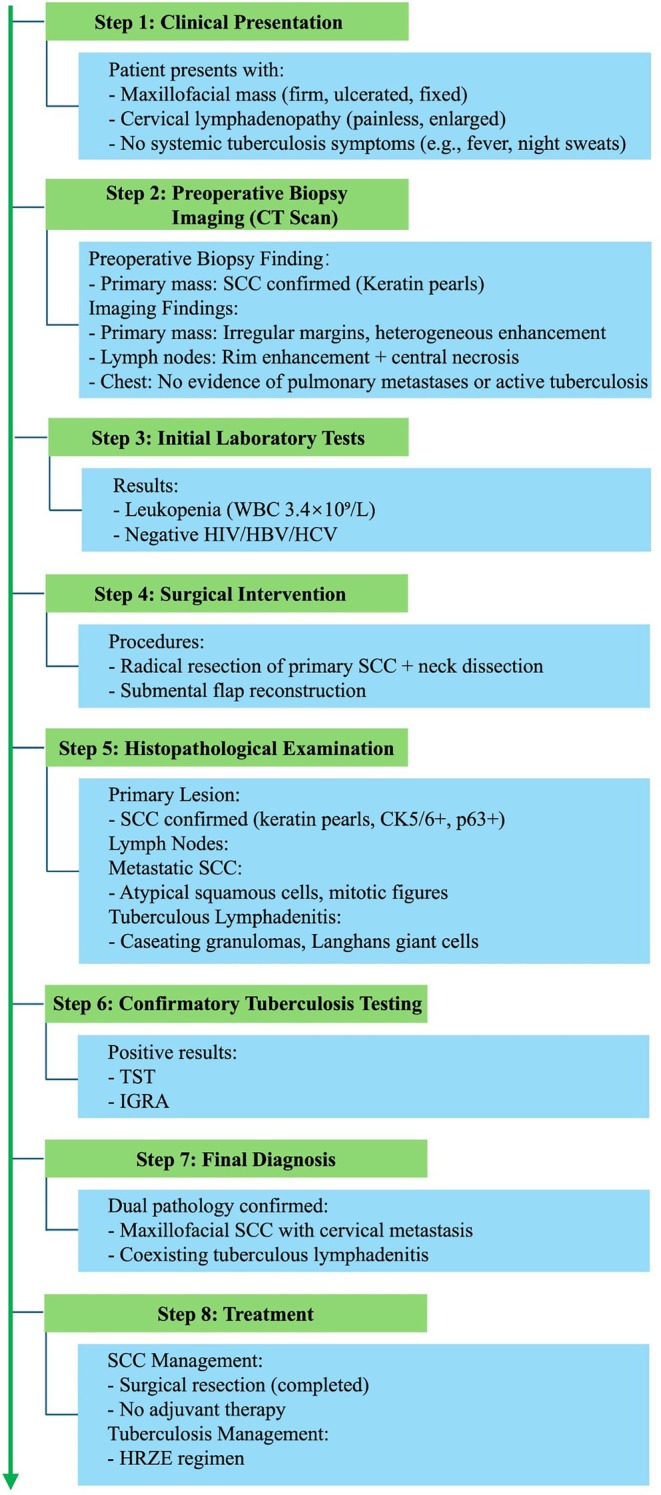
Diagnostic algorithm for cervical lymph node metastasis from maxillofacial SCC complicated by tuberculous lymphadenitis (present case).

Figure 5 presents the diagnostic algorithm for this case, outlining the key diagnostic procedures and highlighting the confirmation process of dual pathology.

Despite thorough counseling regarding the necessity of adjuvant radiotherapy per the National Comprehensive Cancer Network (NCCN) Clinical Practice Guidelines in Oncology due to multiple lymph node metastases, the patient declined this treatment after careful consideration, citing personal reasons and concerns over potential side effects and logistical challenges (such as the inconvenience of traveling to the hospital). Follow‐up at 7 months postoperatively (October 2025) demonstrated well‐healed surgical wounds with no evidence of new lesions on radiographic imaging.

## 7 Discussion

The clinical differentiation of cervical lymph node lesions remains diagnostically challenging, with the primary pathological categories encompassing infectious diseases (bacterial, viral, and tuberculous), congenital head and neck disorders, and malignant neoplasms (both primary and metastatic) [1]. This study reports a 68‐year‐old female patient with right maxillofacial SCC and cervical lymph node metastasis, in whom postoperative pathological examination unexpectedly revealed coexisting metastatic SCC and tuberculous lymphadenitis within regional lymph nodes.

The uniqueness of this case lies in the atypical radiological and clinical manifestations, which presented considerable diagnostic challenges:

1. Radiological findings: CT demonstrated central hypodensity with peripheral thickening and enhancement in the lymph nodes. Although acute‐stage tuberculous lymphadenitis may exhibit homogeneous enhancement on CT imaging, subacute‐stage tuberculous lymphadenitis shares identical features with metastatic SCC, such as central low absorption and irregular peripheral thickening with enhancement. Consequently, radiological differentiation between these two conditions is difficult in this case.
2. Atypical clinical presentation: The patient lacked typical tuberculous toxic symptoms (e.g., low‐grade fever, night sweats) and had no history of tuberculosis, leading clinicians to initially overlook the possibility of coexisting tuberculous lymphadenitis. Literature reports indicate that cervical tuberculous lymphadenitis typically has an insidious onset, primarily manifesting as unilateral, painless, and progressively enlarging lymphadenopathy, which closely resembles metastatic SCC, further complicating clinical diagnosis [7, 8, 9].

Reportedly, the pathogenesis of tuberculous lymphadenitis primarily involves either direct invasion of cervical lymph nodes by inhaled mycobacteria through the tonsils and adenoids, or lymphatic/hematogenous dissemination from primary pulmonary lesions [3, 7]. The coexistence of metastatic SCC and tuberculous lymphadenitis within the same anatomical region has been rarely reported. Potential interaction mechanisms may include the following hypotheses:

1. Given the patient's HIV‐negative status, the possibility of HIV‐related immunodeficiency as a causative factor was essentially ruled out. Moreover, since the coexistence of tuberculous lymphadenitis and metastatic carcinoma within the same regional lymph nodes was not suspected preoperatively, routine CD4+ cell count testing was not performed. However, the observed leukopenia in peripheral blood in this case may, to some extent, indicate tumor microenvironment‐induced immunosuppression, a condition that could potentially promote either newly acquired tuberculosis infection or reactivation of latent tuberculosis [4].
2. The chronic inflammatory microenvironment alterations induced by tuberculous infection (e.g., macrophage activation, cytokine secretion) may also promote cancer cell metastasis or proliferation [5].

Notably, in this case, tuberculous lesions and metastatic carcinoma were located in distinct cervical lymph nodes, suggesting several possibilities. The immune microenvironment may vary significantly among different lymph nodes. Additionally, tumor cells and *Mycobacterium tuberculosis* may exhibit differential lymph node tropism. Furthermore, local immunoregulatory mechanisms could influence the distribution patterns of both pathogens.

The patient received intravenous cefuroxime for surgical site infection prophylaxis, with the initial dose administered 30 min preoperatively and continued for 48 h postoperatively. To mitigate inflammatory edema, intravenous dexamethasone was given for 3 days following surgery. Standard HRZE (isoniazid, rifampin, pyrazinamide, and ethambutol) anti‐tuberculosis therapy was initiated 2 weeks postoperatively. Comprehensive evaluation revealed no evidence of distant malignant metastasis; thus, no antineoplastic agents were administered. No drug–drug interactions were observed during the treatment course.

The coexistence of tuberculous lymphadenitis and metastatic carcinoma within the same regional lymph nodes is clinically uncommon. Although the current study is limited by relatively short‐term follow‐up data (7 months), during which the patient demonstrated favorable recovery (wound healing and no radiologically detectable new lesions), longer‐term follow‐up would provide more robust prognostic evaluation. Furthermore, the patient declined adjuvant radiotherapy recommended by the NCCN guidelines due to personal reasons, including concerns about potential side effects and commuting difficulties. This situation highlights the complexity of implementing standard treatment regimens in clinical practice. Nonetheless, the key clinical significance of this study lies in revealing the substantial risk of misdiagnosis associated with such coexisting pathologies, particularly the potential misinterpretation as pure malignancy, which could critically impact therapeutic decision‐making. Therefore, we emphasize that multidisciplinary collaboration among radiology, pathology, and clinical teams is essential for accurate diagnosis. Of particular note, clinicians should maintain a high index of suspicion for such combined pathological processes when evaluating patients from tuberculosis‐endemic regions.

## 8 Conclusion

This case report describes a rare clinical phenomenon of coexisting metastatic carcinoma and tuberculous lesions within cervical lymph nodes of a patient with maxillofacial SCC. These findings suggest that clinicians should adopt a broader diagnostic perspective when evaluating lymph node lesions in head and neck cancer patients, with careful consideration given to the possibility of concurrent multiple pathological conditions.

## Author Contributions

Xierzhati Tuerxun and Meiheriban Tuerhong drafted the manuscript. Zainure Wubulihasimu prepared the figures. Xierzhati Tuerxun, Meiheriban Tuerhong, Zainure Wubulihasimu, and Baihetiyaer Yimin participated in patient treatment and data collection. Kai Liu and Tuerdi Maimaitituxun designed the treatment protocol, performed clinical interventions, supervised the study, and revised the manuscript. All authors critically reviewed the final draft.

## Funding

The authors have nothing to report.

## Ethics Statement

The study was approved by the Institutional Review Board of Kashi Prefecture Second People's Hospital (Approval No. [2025] Lunkeshen‐25).

## Consent

Informed consent was obtained from the patient prior to the study.

## Conflicts of Interest

The authors declare no conflicts of interest.

## Data Availability

The data that support the findings of this study are available from the corresponding author upon reasonable request.
